# The association of plasma homocysteine levels with short-term mortality in sepsis patients: A meta-analysis

**DOI:** 10.17305/bb.2024.11259

**Published:** 2024-10-29

**Authors:** Xinxing Lu, Xueyan Yuan, Yali Chao, Xiao Wu, Airan Liu

**Affiliations:** 1 Jiangsu Provincial Key Laboratory of Critical Care Medicine, Department of Critical Care Medicine, Zhongda Hospital, School of Medicine, Southeast University, Nanjing, Jiangsu, People’s Republic of China

**Keywords:** Homocysteine, hyperhomocysteinemia, sepsis, mortality, meta-analysis

## Abstract

The association between plasma homocysteine (Hcy) levels and short-term mortality in sepsis patients remains unclear. This meta-analysis aimed to clarify this potential relationship. Following PRISMA 2020 and Cochrane Handbook guidelines, we conducted a comprehensive literature search in the PubMed, Embase, and Web of Science databases up to June 24, 2024. We included cohort studies that assessed the association between plasma Hcy levels and all-cause mortality in adult sepsis patients. Standardized mean differences (SMDs) and odds ratios (ORs) with 95% confidence intervals (CIs) were calculated using a random-effects model to account for potential heterogeneity. Nine cohort studies involving 771 sepsis patients were included. Overall, no significant difference in plasma Hcy levels was observed between survivors and non-survivors (SMD: −0.23, 95% CI: −0.84 to 0.37, *P* ═ 0.45), with substantial heterogeneity (*I^2^* ═ 86%). Subgroup analysis revealed lower plasma Hcy levels among survivors in Chinese patients (SMD: −1.56, 95% CI: −1.98 to −1.13, *P* < 0.001) but not in non-Asian patients. Plasma Hcy levels were not significantly associated with all-cause mortality (OR per 1-unit increment: 1.03, 95% CI: 0.95–1.11, *P* ═ 0.51), with notable heterogeneity (*I^2^* ═ 72%). However, a significant association was found in Chinese patients (OR: 1.09, 95% CI: 1.03–1.15, *P* ═ 0.003), but not in non-Asian patients. In conclusion, plasma Hcy levels were not generally associated with short-term mortality in sepsis patients. However, significant associations were observed in Chinese patients, suggesting potential ethnic differences that warrant further investigation.

## Introduction

Sepsis, a life-threatening organ dysfunction caused by a dysregulated host response to infection, remains a major global health concern [1, 2]. In 2017, the disease was responsible for an estimated 48.9 million cases and 11 million sepsis-related deaths annually [3], making it one of the leading causes of mortality worldwide [4]. Patients with sepsis often experience rapid progression to severe conditions, including septic shock and multi-organ failure, which contributes to high short-term mortality rates [5]. Despite advancements in medical care, the prognosis for sepsis remains poor, especially in the early stages of the illness [6, 7]. Therefore, identifying reliable risk factors for short-term mortality in sepsis patients is essential to improve clinical outcomes and guide therapeutic interventions.

Biomarkers play a critical role in the prognostic stratification of sepsis patients [8]. Among various potential biomarkers, homocysteine (Hcy) has garnered particular interest due to its involvement in oxidative stress, endothelial dysfunction, and inflammation—key mechanisms in sepsis pathophysiology [9, 10]. Hcy is a sulfur-containing amino acid formed during methionine metabolism [11]. Elevated plasma Hcy levels, known as hyperhomocysteinemia (HHcy), have been linked to numerous pathological conditions, including cardiovascular diseases, neurodegenerative disorders, and renal failure [12, 13]. Hcy metabolism involves remethylation to methionine or transsulfuration to cysteine, both of which require vitamins B6, B12, and folate as cofactors [14]. Several mechanisms may explain the link between high Hcy levels and increased mortality in critically ill patients, including those with sepsis, such as enhanced oxidative stress, impaired endothelial function, promotion of a pro-thrombotic state, and an exacerbated inflammatory response [15].

Evaluating Hcy as a prognostic marker in sepsis is particularly relevant given potential ethnic differences in Hcy metabolism. Factors, such as dietary habits and genetic polymorphisms, can influence plasma Hcy levels [16, 17]. For example, Asian populations, particularly in China, often have lower plasma folate levels compared to Western populations, which may impact Hcy metabolism [18, 19]. Additionally, genetic variations, such as those in the methylenetetrahydrofolate reductase (*MTHFR*) gene, play a significant role in Hcy metabolism and vary between ethnic groups [20]. The *MTHFR* C677T polymorphism, associated with increased Hcy, is more prevalent in Asian populations compared to non-Asian populations [21]. These differences underscore the importance of considering ethnicity when evaluating the prognostic role of Hcy in sepsis.

Previous studies examining the association between plasma Hcy levels and short-term mortality in sepsis patients have produced inconsistent results. While some studies report an association between high plasma Hcy levels and increased mortality risk [22, 23], others do not [24–30]. To address these inconsistencies, this meta-analysis aimed to clarify the association between plasma Hcy levels and short-term mortality in sepsis patients. We systematically reviewed and synthesized data from cohort studies to determine whether elevated plasma Hcy levels at sepsis diagnosis are associated with an increased risk of short-term mortality. Additionally, we explored whether this association varies between Asian and non-Asian populations, considering the influence of dietary factors and genetic polymorphisms on Hcy metabolism.

## Materials and methods

This study adhered to the PRISMA 2020 guidelines [31] and the Cochrane Handbook for Systematic Reviews and Meta-analyses [31] for conducting meta-analyses, covering study design, data collection, statistical analysis, and result interpretation. The protocol for this systematic review and meta-analysis is registered on the Open Science Framework (https://osf.io/5mr3j).

### Literature search

To identify relevant studies, we searched the PubMed, Embase, and Web of Science databases using a comprehensive array of search terms: (1) “homocysteine” OR “hyperhomocysteinemia” OR “Hcy” OR ”HHcy” OR “2-amino-4-mercaptobutyric acid”; and (2) “sepsis” OR “septicemia” OR “septic.” We limited the search to studies involving human subjects and included only full-text articles in English or Chinese from peer-reviewed journals. Detailed search syntax for each database is provided in Supplemental File 1. Additionally, we manually reviewed references from relevant original and review articles to identify additional studies. The literature search covered database inception up to June 24, 2024.

### Inclusion and exclusion criteria

Studies were included if they met the following criteria: (1) cohort studies, including both prospective and retrospective designs; (2) involved adult patients with a confirmed sepsis diagnosis; (3) measured plasma Hcy at enrollment and analyzed it as an exposure; (4) had all-cause mortality as the primary outcome in sepsis patients; and (5) reported either the difference in plasma Hcy between survivors and non-survivors or the association between plasma Hcy and all-cause mortality. Studies were excluded if they were reviews, editorials, meta-analyses, preclinical studies, cross-sectional studies, studies without sepsis patients, studies lacking baseline plasma Hcy evaluation, or studies that did not report all-cause mortality. In cases of overlapping populations across studies, only the study with the largest sample size was included in the meta-analysis.

### Study quality evaluation

The literature search, study identification, quality assessment, and data extraction were conducted independently by two authors, with any disagreements resolved through discussion with the corresponding author. Study quality was assessed using the Newcastle–Ottawa Scale (NOS) [32], which evaluates selection, control of confounders, and outcome measurement, with scores ranging from 1 to 9, where 9 indicates the highest quality.

### Data extraction

Data extracted for analysis included study details (author, year, country, and design), participant characteristics (diagnosis criteria for sepsis, sample size, age, and sex), timing and methods for plasma Hcy measurement, follow-up duration, number of deaths during follow-up, and reported outcomes. Additionally, we collected variables adjusted or matched in assessing the association between plasma Hcy and all-cause mortality risk in sepsis patients.

### Statistical analysis

We used the standardized mean difference (SMD) and corresponding 95% confidence interval (CI) to summarize plasma Hcy levels at enrollment between survivors and non-survivors, and the odds ratio (OR) and corresponding 95% CI to evaluate the association between plasma Hcy (per 1-unit increase) and all-cause mortality risk. OR values and their standard errors were computed from 95% CIs or *P* values and logarithmically transformed for variance stabilization. Heterogeneity was assessed using the Cochrane *Q* test and *I^2^* statistic [33], with *I^2^* > 50% indicating substantial heterogeneity. A random-effects model was applied to account for study variability [31].

**Figure 1.**
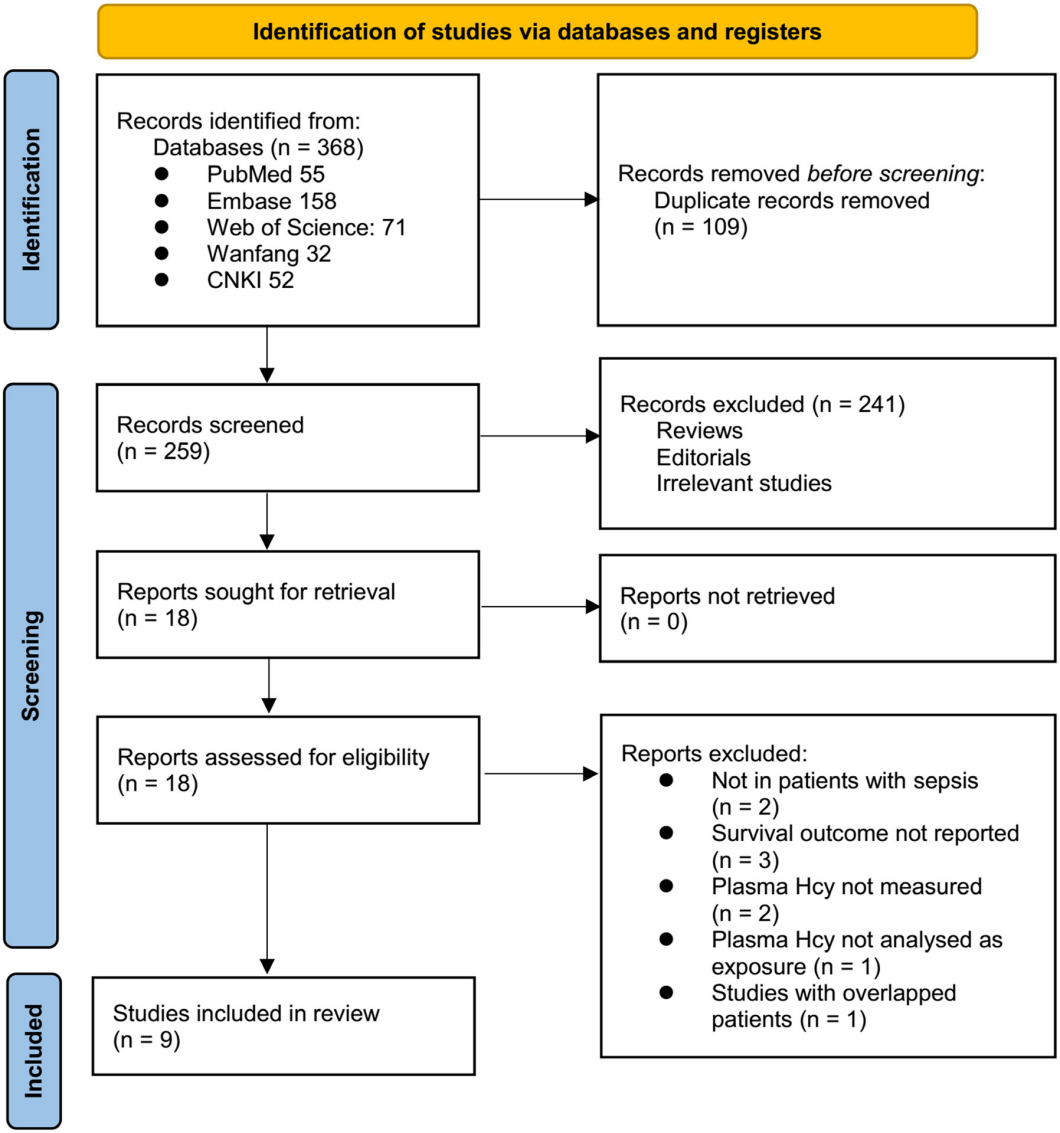
PRISMA flowchart of database search and study identification.

To assess the robustness of findings, we conducted a sensitivity analysis by sequentially excluding individual studies. For the primary outcome, predefined subgroup analyses explored the effects of various factors, such as geographic region (Asian or non-Asian), sepsis diagnostic criteria, mean age, proportion of male participants, follow-up duration, and analytical models (multivariate or univariate). Subgroups for continuous variables were defined using median values. For the secondary outcome, we performed a subgroup analysis based on geographic region (Asian or non-Asian). Finally, publication bias was evaluated using funnel plots and visual inspection for asymmetry, supplemented by Egger’s regression test [34]. All analyses were conducted using RevMan (Version 5.1; Cochrane Collaboration, Oxford, UK) and Stata software (Version 12.0; Stata Corporation, College Station, TX, USA).

## Results

### Study inclusion

The study inclusion process is illustrated in Figure 1. Initially, 368 potentially relevant records were identified from the five databases searched, with 109 records excluded due to duplication. A subsequent screening of titles and abstracts led to the exclusion of an additional 241 studies, primarily because they did not align with the objectives of this meta-analysis. The full texts of the remaining 18 records were reviewed by two independent authors, resulting in the exclusion of nine more studies for various reasons, as detailed in Figure 1. Ultimately, nine cohort studies met the inclusion criteria and were included in the quantitative analysis [22–30].

### Overview of study characteristics

Table 1 summarizes the characteristics of the studies included in this meta-analysis. In total, four prospective cohort studies [25, 27, 28, 30] and five retrospective cohort studies [22–24, 26, 29] were included, published between 2000 and 2023 and conducted in Austria, Brazil, Greece, the United States, China, and Italy. These studies involved 771 adult patients diagnosed with sepsis according to Sepsis-1.0 [24, 25, 27], Sepsis-2.0 [26], or Sepsis-3.0 criteria [22, 23, 28–30]. Plasma Hcy was measured within 4–48 h after sepsis diagnosis using various methods, including high-performance liquid chromatography (HPLC) [24–26, 30], competitive particle-enhanced immunonephelometry [27, 29], liquid chromatography coupled with positive-electron ion spray mass spectrometry [28], enzymatic methods via automated biochemical analyzers [22], or radioimmunoassay [23].

**Table 1:** Characteristics of the included studies

**Study**	**Location**	**Design**	**Diagnosis of sepsis**	**No. of patients**	**Mean age (years)**	**Men (%)**	**Timing of Hcy measuring**	**Methods of Hcy measuring**	**Follow-up duration**	**Patients died**	**Outcomes reported**	**Variables matched or adjusted**
Stoiser et al., 2000	Austria	RC	Sepsis–1.0	14	63	50	Within 24 h after diagnosis of sepsis	HPLC	30 days	7	Difference of serum Hcy	None
Neto et al., 2010	Brazil	PC	Sepsis–1.0	21	43.9	52.4	Within 24 h after diagnosis of sepsis	HPLC	28 days	6	Difference of serum Hcy	None
Tsantes et al., 2010	Greece	PC	Sepsis–1.0	102	61.9	66.7	Within 48 h after diagnosis of sepsis	CPEI	28 days	41	Difference of serum Hcy, and OR per 1-unit increment of Hcy	Age, APACHE II score, FVL or MTHFR genotypes, plasma protein C, SCr, vitamin B12 and folate levels
Ploder et al., 2010	Austria	RC	Sepsis–2.0	18	45.2	77.8	Within 24 h after diagnosis of sepsis	HPLC	28 days	7	Difference of serum Hcy	None
Wexler et al., 2018	USA	PC	Sepsis–3.0	109	62	57	Within 48 h after diagnosis of sepsis	LC-ESI-MS/MS MRM	In-hospital	31	Difference of serum Hcy	None
Liu et al., 2021	China	RC	Sepsis–3.0	352	58.8	59.4	At the diagnosis of sepsis	CPEI	28 days	49	OR per 1-unit increment of Hcy	Age, sex, SOFA score, CRP, SCr, and PCT
Chen et al., 2021	China	RC	Sepsis–3.0	60	79.1	51.7	Within 4 h after diagnosis of sepsis	Enzymatic method by automated biochemical analyzer	28 days	22	Difference of serum Hcy, and OR per 1-unit increment of Hcy	Age, sex, and SOFA score
Belli et al., 2022	Italy	PC	Sepsis–3.0	35	59	60	Within 24 h after diagnosis of sepsis	HPLC	In-hospital	15	Difference of serum Hcy, and OR per 1-unit increment of Hcy	None
Wang et al., 2023	China	RC	Sepsis–3.0	60	48.4	40	Within 24 h after diagnosis of sepsis	RIA	28 days	34	Difference of serum Hcy	None

Hcy: Homocysteine; RC: Retrospective cohort; PC: Prospective cohort; HPLC: High-performance liquid chromatography; CPEI: Competitive particle-enhanced immunonephelometry; LC-ESI-MS/MS MRM: Liquid chromatography coupled with positive-electron ion spray mass spectrometry in multiple reaction monitoring mode; RIA: Radioimmunoassay; OR: Odds ratio; APACHE II: The acute physiology and chronic health evaluation II; FVL: Factor V leiden; MTHFR: Methylenetetrahydrofolate reductase; SCr: Serum creatinine; SOFA: Sequential organ failure assessment; CRP: C-reactive protein; PCT: Procalcitonin.

Follow-up periods varied, with two studies following patients until hospital discharge [28, 30] and the others following them for 28 or 30 days [22–27, 29]. A total of 212 patients (27.5%) died during follow-up. Eight studies reported plasma Hcy levels for survivors and non-survivors [22–28, 30], and four studies reported the OR for all-cause mortality per 1-unit increment of plasma Hcy [22, 27, 29, 30]. Univariate analysis was used in six studies to evaluate the association between plasma Hcy and mortality in sepsis patients [23–26, 28, 30], while the remaining three studies used multivariate analysis [22, 27, 29]. The NOS scores for included studies ranged from 6 to 9, indicating moderate to good quality (Table 2).

**Table 2:** Study quality evaluation via the Newcastle–Ottawa scale

**Study**	**Representativeness of the exposed cohort**	**Selection of the non-exposed cohort**	**Ascertainment of exposure**	**Outcome not present at baseline**	**Control for age**	**Control for other confounding factors**	**Assessment of outcome**	**Enough long follow-up duration**	**Adequacy of follow-up of cohorts**	**Total**
Stoiser et al., 2000	0	1	1	1	0	0	1	1	1	6
Neto et al., 2010	1	1	1	1	0	0	1	1	1	7
Tsantes et al., 2010	1	1	1	1	1	1	1	1	1	9
Ploder et al., 2010	0	1	1	1	0	0	1	1	1	6
Wexler et al., 2018	1	1	1	1	1	1	1	1	1	9
Liu et al., 2021	0	1	1	1	1	1	1	1	1	8
Chen et al., 2021	0	1	1	1	1	1	1	1	1	8
Belli et al., 2022	1	1	1	1	0	0	1	1	1	7
Wang et al., 2023	0	1	1	1	0	0	1	1	1	6

### Difference in plasma Hcy at enrollment between survivors and non-survivors

**Figure 2.**
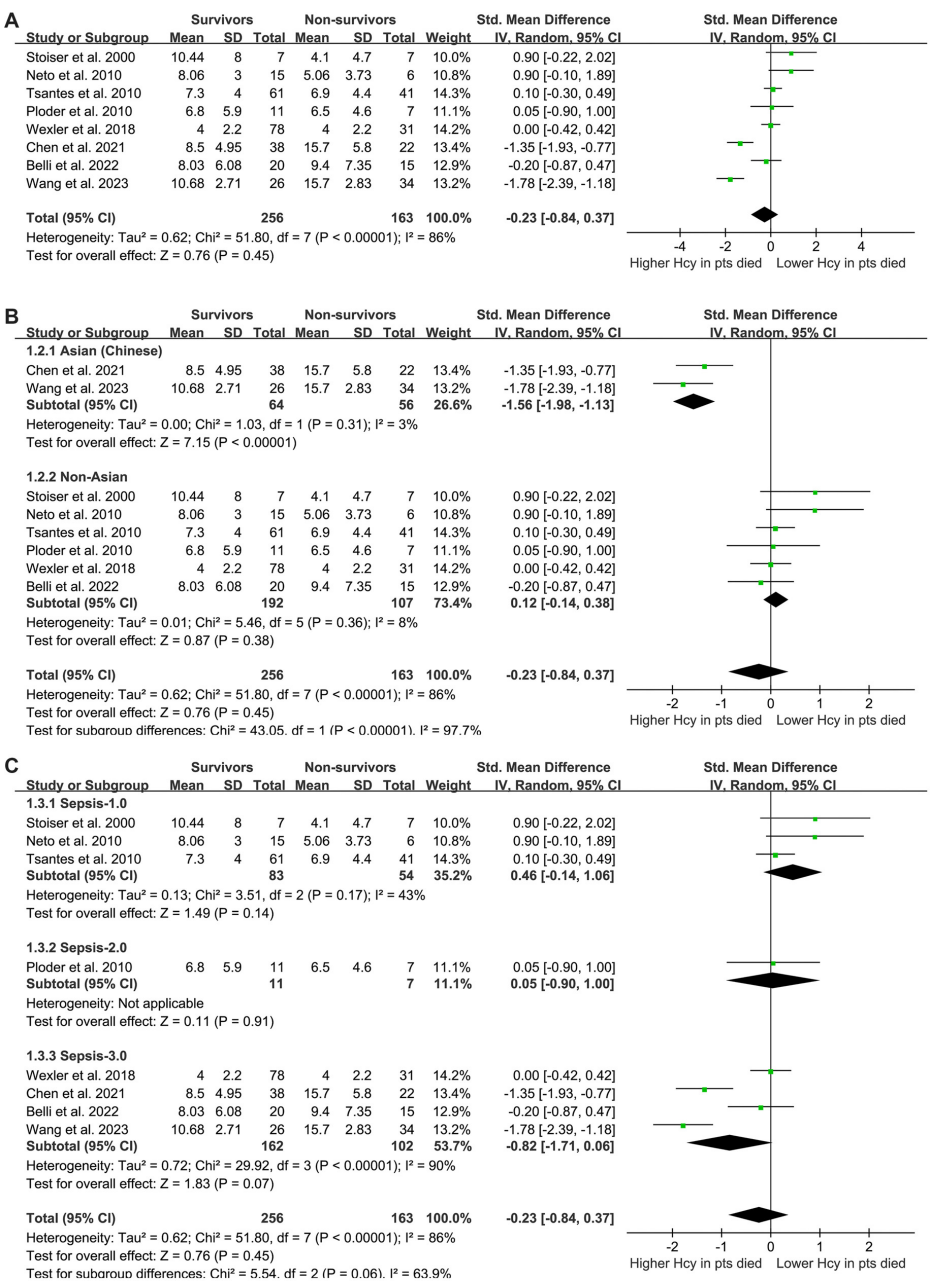
**The forest plots for this meta-analysis illustrate the differences in plasma homocysteine levels at enrollment between survivors and non-survivors of sepsis, divided into several analyses.** (A) Overall meta-analysis; (B) Subgroup analysis according to the study countries; (C) Subgroup analysis according to the diagnostic criteria for sepsis.

The pooled results from eight studies [22–28, 30] suggested no significant difference in plasma Hcy levels at enrollment between survivors and non-survivors of sepsis (SMD: −0.23, 95% CI: −0.84 to 0.37, *P* ═ 0.45; Figure 2A) with significant heterogeneity (*I^2^* ═ 86%). Sensitivity analysis, which involved excluding one study at a time, produced similar results (SMD: −0.37 to −0.03, *P* > 0.05 for all).

**Figure 3.**
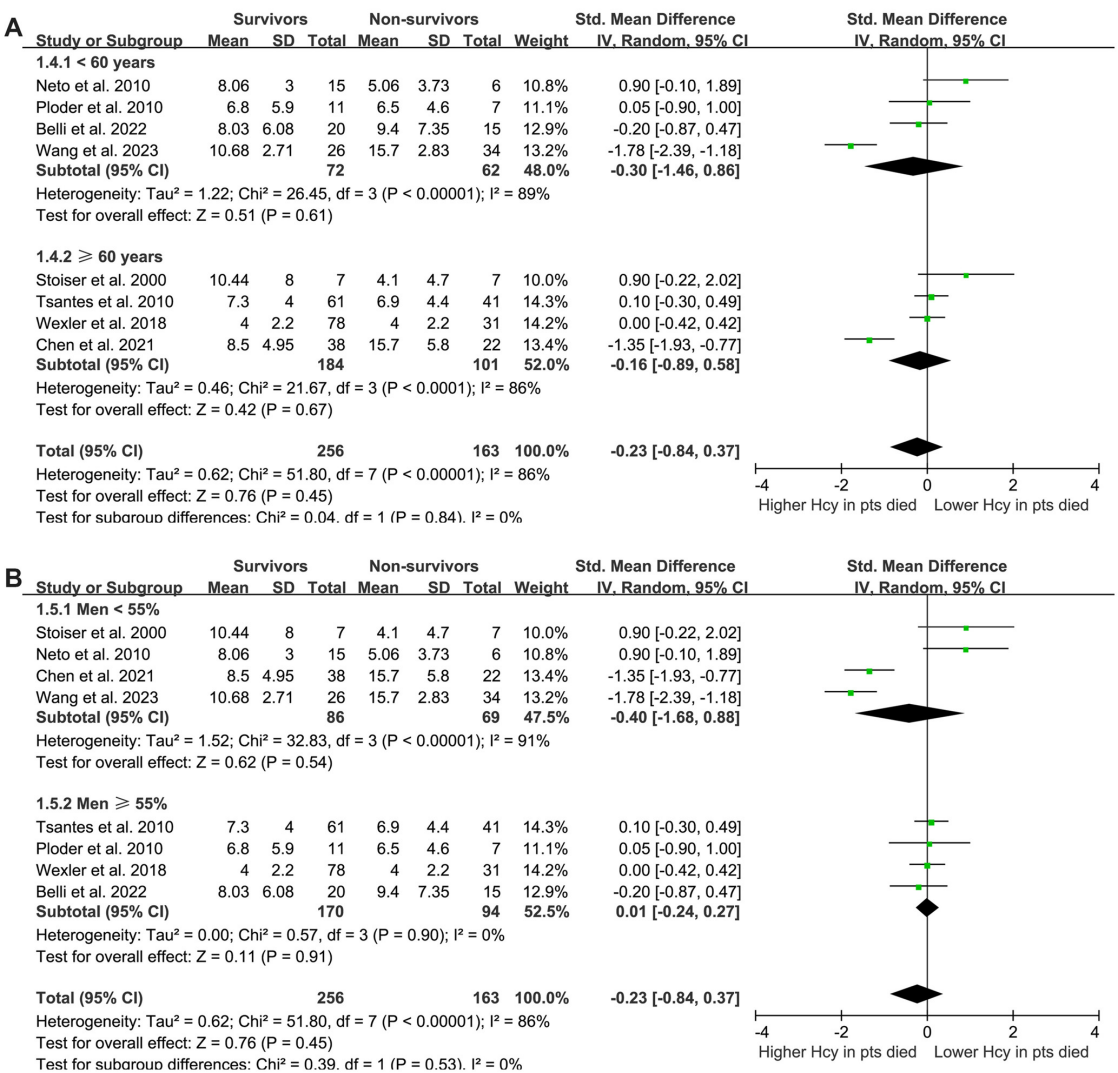
**The forest plots for this meta-analysis examine the difference in plasma homocysteine levels at enrollment between survivors and non-survivors of sepsis, broken down by specific subgroup analyses.** (A) Subgroup analysis according to the mean age of the patients; (B) Subgroup analysis according to the proportion of men.

**Figure 4.**
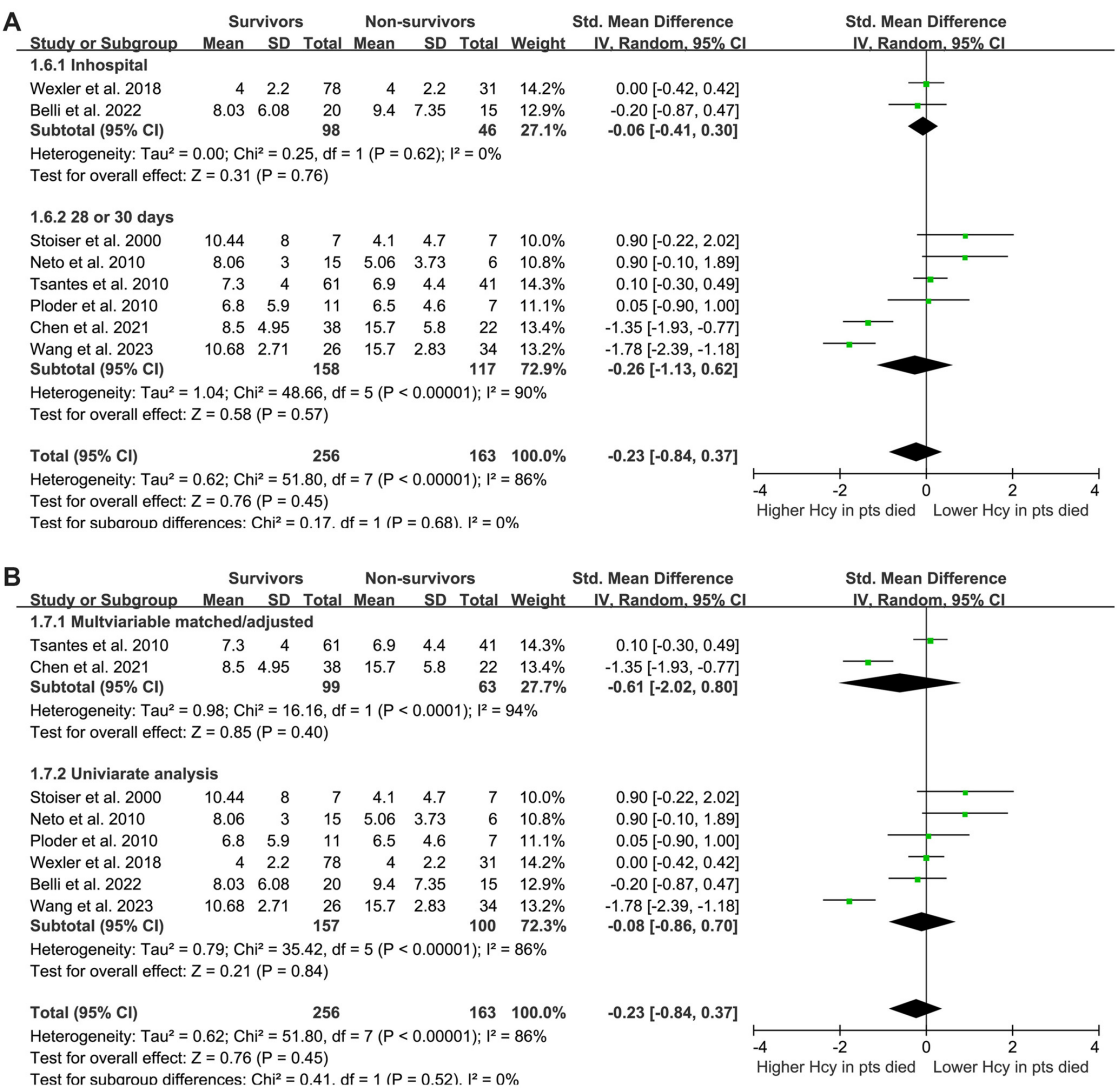
**The forest plots for this meta-analysis illustrate the differences in plasma homocysteine levels at enrollment between survivors and non-survivors of sepsis, divided into several analyses.** (A) Subgroup analysis according to the follow-up durations; (B) Subgroup analysis according to the analytic models.

Interestingly, subgroup analysis revealed a lower plasma Hcy level in survivors compared to non-survivors among Chinese patients with sepsis (SMD: −1.56, 95% CI: −1.98 to −1.13, *P* < 0.001; *I^2^* ═ 3%), but not among non-Asian patients (SMD: 0.12, 95% CI: −0.14 to 0.38, *P* ═ 0.38; *I^2^* ═ 8%), which fully explained the source of heterogeneity (*P* for subgroup difference < 0.001; Figure 2B). These results suggest that while plasma Hcy levels may not differentiate between survivors and non-survivors in a general sepsis population, ethnic differences in Hcy metabolism may influence outcomes in sepsis. Further subgroup analyses showed that differences in sepsis diagnostic criteria (*P* for subgroup difference ═ 0.06; Figure 2C), mean patient age (*P* ═ 0.84; Figure 3A), proportion of men (*P* ═ 0.53; Figure 3B), follow-up duration (*P* ═ 0.68; Figure 4A), and analytical model (*P* ═ 0.52; Figure 4B) did not affect the meta-analysis results.

### OR for the association between plasma Hcy at enrollment and all-cause mortality

**Figure 5.**
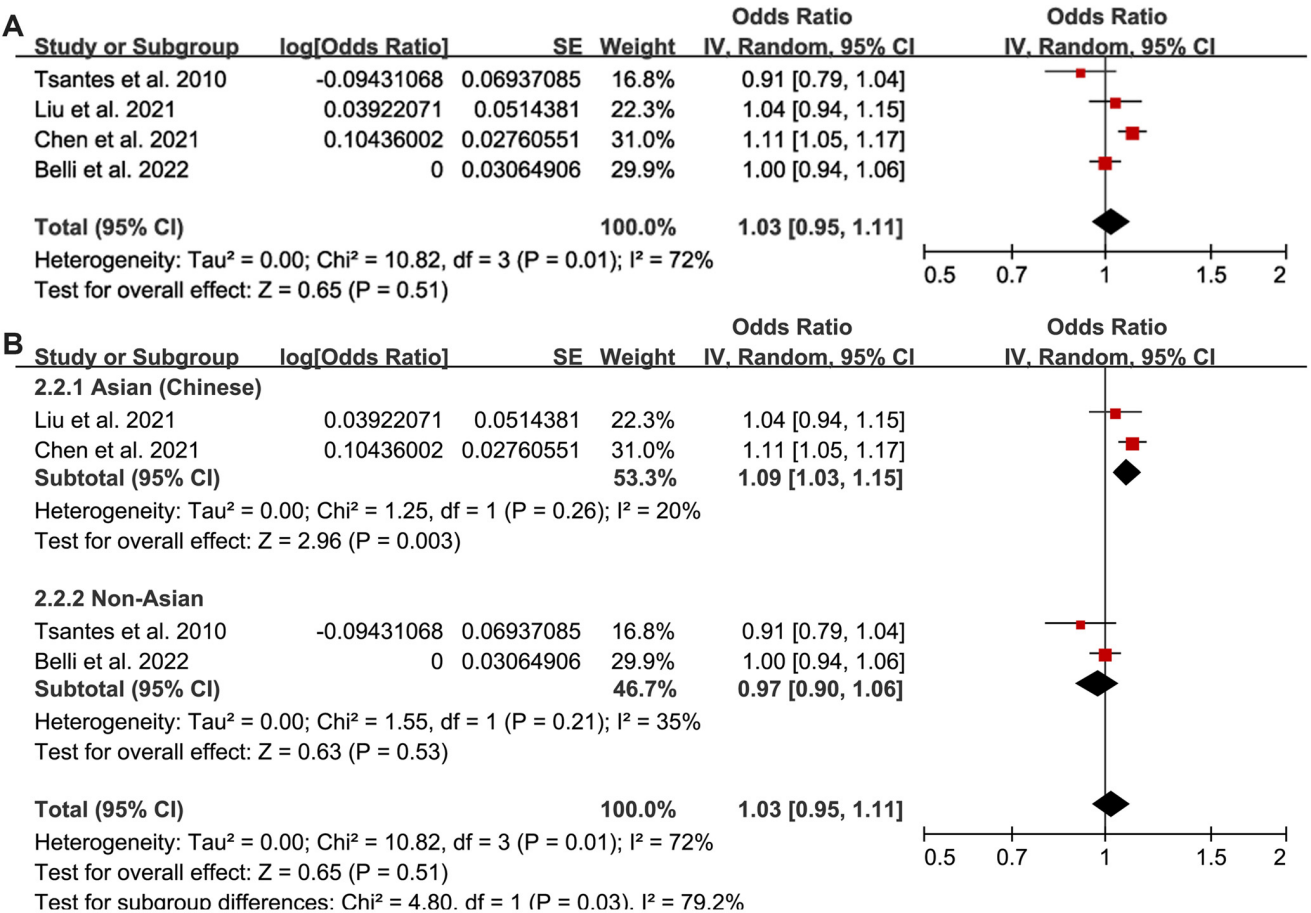
**The forest plots for this meta-analysis display the odds ratios for the association between plasma homocysteine levels (per 1-unit increment) at enrollment and all-cause mortality in patients with sepsis.** (A) Overall meta-analysis; (B) Subgroup analysis according to the study countries.

The pooled results from four studies [22, 27, 29, 30] indicated that high plasma Hcy at enrollment was not associated with an increased risk of death in patients with sepsis (OR per 1-unit increment of Hcy ═ 1.03, 95% CI: 0.95–1.11, *P* ═ 0.51; *I^2^* ═ 72%; Figure 5A). Sensitivity analysis by omitting one study at a time yielded consistent results (OR: 1.00–1.05, *P* > 0.05 for all).

Subgroup analysis showed that plasma Hcy was significantly associated with all-cause mortality risk in Chinese sepsis patients (OR per 1-unit increment of Hcy ═ 1.09, 95% CI: 1.03–1.15, *P* ═ 0.003; *I^2^* ═ 20%), but not in non-Asian patients (OR per 1-unit increment of Hcy ═ 0.97, 95% CI: 0.90–1.06, *P* ═ 0.53; *I^2^* ═ 35%). The difference between subgroups was statistically significant (*P* ═ 0.03; Figure 5B). These findings highlight the potential prognostic value of plasma Hcy specifically in Chinese sepsis patients, suggesting that ethnicity may play a role in the prognostic implications of Hcy levels.

### Publication bias

**Figure 6.**
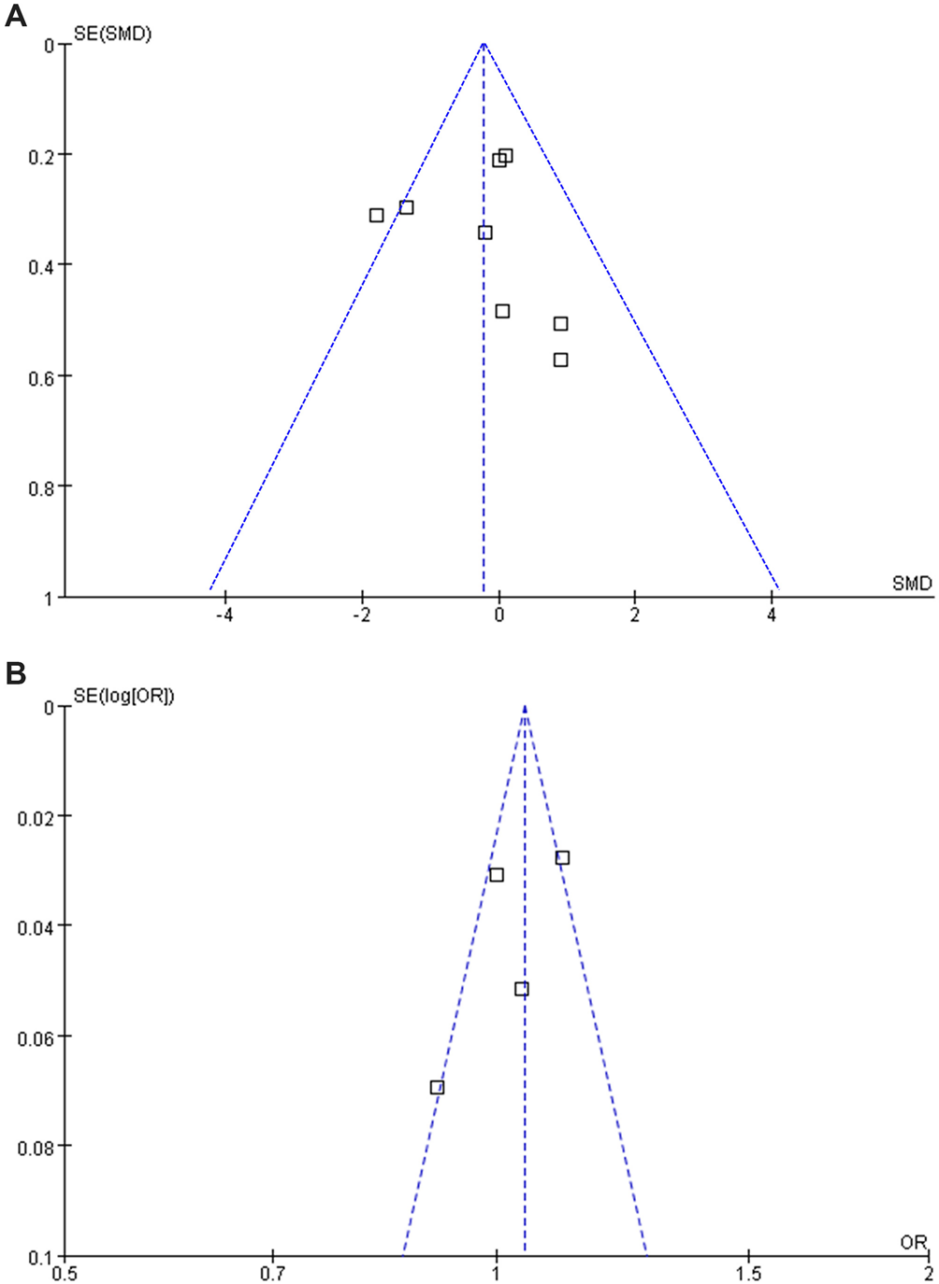
**Funnel plots for meta-analyses.** (A) Funnel plots for the meta-analysis of the difference of plasma Hcy between survivors and non-survivors of sepsis; (B) Funnel plots for the meta-analysis of the OR for the association between plasma Hcy (per 1-unit increment) and mortality of patients with sepsis. Hcy: Homocysteine; OR: Odds ratio.

Funnel plots for the meta-analyses of plasma Hcy differences between survivors and non-survivors, and for the OR for the association between plasma Hcy and mortality, are shown in Figure 6A and 6B, respectively. Both plots appeared symmetrical on visual inspection, indicating a low risk of publication bias. Egger’s regression test for the analysis of plasma Hcy differences between survivors and non-survivors also indicated a low risk of publication bias (*P* ═ 0.41). Egger’s test for the OR analysis was not performed due to the limited number of studies (*n* ═ 4).

## Discussion

This meta-analysis aimed to clarify the association between Hcy levels and short-term mortality in patients with sepsis. Our findings suggest that, overall, there is no significant difference in plasma Hcy levels between survivors and non-survivors of sepsis. However, subgroup analysis revealed that elevated Hcy levels were significantly associated with higher mortality risk in Chinese patients, but not in non-Asian patients. These results highlight that ethnic differences in Hcy metabolism should be considered when assessing its role in predicting outcomes in sepsis.

The lack of a significant overall association between plasma Hcy levels and short-term mortality in sepsis patients may be attributed to several factors. First, heterogeneity in study designs, patient populations, and methods for measuring Hcy could have contributed to the variability in results. Second, the timing of Hcy measurement after sepsis diagnosis varied across studies, potentially influencing observed Hcy levels. Third, varying sepsis diagnostic criteria in the included studies may also explain some of the differences in findings. Despite these limitations, our subgroup analysis indicated a significant association between increased Hcy levels and increased mortality in Chinese patients with sepsis, but not in non-Asian patients.

The mechanisms underlying the observed ethnic differences in the association between Hcy and sepsis mortality are not fully understood, but several factors may be involved. One possible explanation is the higher prevalence of the *MTHFR* C677T polymorphism in Asian populations, including Chinese, which is associated with elevated Hcy levels [35, 36]. Individuals with this genetic variant have reduced MTHFR enzyme activity, leading to impaired Hcy metabolism and higher plasma Hcy levels. Additionally, dietary differences, such as lower folate intake in some Asian populations, could further exacerbate Hcy elevation [37]. Folate, along with vitamins B6 and B12, is a crucial cofactor in the remethylation and transsulfuration pathways of Hcy metabolism [38]. Lower intake of these vitamins may contribute to higher Hcy levels and, consequently, increased mortality risk in sepsis patients.

Another potential mechanism linking high Hcy levels to increased mortality in sepsis involves Hcy-induced endothelial dysfunction [39] and oxidative stress [40]. Elevated Hcy has been shown to promote the production of reactive oxygen species (ROS), leading to oxidative damage and impaired endothelial function. In the context of sepsis, where oxidative stress and endothelial dysfunction are already prominent, elevated Hcy levels could exacerbate these pathological processes, contributing to worse outcomes [41]. Moreover, Hcy has been implicated in promoting a pro-thrombotic state, which could further complicate the already high risk of thrombotic events in sepsis patients [42]. These combined effects of Hcy on endothelial function, oxidative stress, and thrombosis may help explain the observed association between high Hcy levels and increased mortality in Chinese patients with sepsis.

The strengths of this meta-analysis include a comprehensive literature search, adherence to PRISMA and Cochrane guidelines, and the use of rigorous inclusion and exclusion criteria. Including both prospective and retrospective cohort studies enhances the generalizability of the findings. Additionally, the subgroup analysis allowed us to explore potential sources of heterogeneity and identify significant ethnic differences in the association between Hcy and sepsis mortality. However, several limitations should be acknowledged. First, the included studies varied in their methods for measuring plasma Hcy, which may have introduced measurement bias. Second, the timing of Hcy measurement after sepsis diagnosis differed across studies, potentially affecting observed levels. Third, the relatively small number of studies included in the meta-analysis, particularly for the subgroup analysis, limits the robustness of the findings, which may be the most impactful limitation. Fourth, the observational nature of the included studies precludes establishing causality between elevated Hcy levels and increased sepsis mortality. Finally, unmeasured confounding factors, such as variations in treatment protocols and sepsis severity, could have influenced the results.

From a clinical perspective, our findings suggest that plasma Hcy levels may have prognostic value in certain populations, particularly Chinese patients with sepsis. This highlights the potential importance of personalized medicine approaches that consider genetic and dietary factors when evaluating biomarkers for disease prognosis [43]. Clinicians should be aware of potential ethnic differences in Hcy metabolism and consider these factors when interpreting Hcy levels in sepsis patients. Routine assessment of plasma Hcy levels in sepsis patients, particularly those of Asian descent, could provide valuable prognostic information and help guide therapeutic decisions. Implementing routine Hcy assessments could lead to a more personalized approach to patient care, especially for Asian patients. Clinicians might consider Hcy levels alongside other clinical parameters to better stratify risk and tailor therapeutic interventions. However, given the limitations of our findings and the need for further research, it would be premature to establish specific treatment protocols based solely on Hcy levels. Future studies should aim to further clarify the mechanisms underlying the observed ethnic differences in the association between Hcy and sepsis mortality. Large-scale, multi-ethnic cohort studies with standardized methods for measuring plasma Hcy (such as HPLC) and comprehensive data on genetic and dietary factors are needed to confirm and extend our findings. Randomized controlled trials investigating the effects of interventions aimed at lowering plasma Hcy levels, such as folate and B-vitamin supplementation, on sepsis outcomes could provide valuable insights into the potential therapeutic benefits of targeting Hcy metabolism in this patient population.

## Conclusion

In conclusion, this meta-analysis found no overall significant association between plasma Hcy levels and short-term mortality in patients with sepsis. However, a significant association was observed in Chinese patients, suggesting that high Hcy levels may be a useful prognostic marker in this population. The potential ethnic differences in Hcy metabolism and their impact on sepsis outcomes warrant further investigation. These findings underscore the importance of personalized approaches in the evaluation and management of sepsis, considering genetic and dietary factors that may influence biomarker levels and disease prognosis.

## Supplemental data

**Detailed search strategy for each database**

**PubMed: 55**

(“homocysteine”[MeSH Terms] OR “homocysteine” OR “hyperhomocysteinemia” OR “Hcy” OR ”HHcy” OR “2-amino-4-mercaptobutyric acid”) AND (“sepsis”[MeSH Terms] OR “sepsis” OR “septicemia” OR “septic”)

**Embase: 158**

(‘homocysteine’/exp OR ‘homocysteine’ OR ‘hyperhomocysteinemia’ OR ‘hcy’ OR ‘hhcy’ OR ‘.amino 4 mercaptobutyric acid’) AND (‘sepsis’/exp OR ‘sepsis’ OR ‘septicemia’ OR ‘septic’) AND [humans]/lim AND [clinical study]/lim AND [embase]/lim

**Web of Science: 71**

TS ═ (“homocysteine” OR “hyperhomocysteinemia” OR “Hcy” OR “HHcy” OR “2-amino-4-mercaptobutyric acid”) AND TS ═ (“sepsis” OR “septicemia” OR “septic”)

**Wanfang: 32**

(“” OR ”” OR “Hcy” OR “HHcy” OR “2–4-”) AND (“” OR “” OR “”)

**Translation in English:** (“homocysteine” OR “hyperhomocysteinemia” OR “Hcy” OR “HHcy” OR “2-amino-4-mercaptobutyric acid”) AND (“sepsis” OR “septicemia” OR “septic”)

**CNKI: 52**

(“” OR “” OR “Hcy” OR “HHcy” OR “2–4-”) AND (“” OR “” OR “”)

**Translation in English:** (“haomocysteine” OR “hyperhomocysteinemia” OR “Hcy” OR “HHcy” OR “2-amino-4-mercaptobutyric acid”) AND (“sepsis” OR “septicemia” OR “septic”)

## Data Availability

All the data generated during the study was within the manuscript and the supplemental data.
